# Riparian Landscape Change: A Spatial Approach for Quantifying Change and Development of a River Network Restoration Model

**DOI:** 10.1007/s00267-024-02025-w

**Published:** 2024-08-20

**Authors:** Martin Stieger, Paul Mckenzie

**Affiliations:** School of Geography and Environmental Sciences, Ulster University, Cromore Road, Coleraine Northern Ireland, BT52 1SA Coleraine, UK

**Keywords:** GIS, River Restoration, Riparian Zone, Restoration Priority Model, Landscape Metrics, Planning

## Abstract

Natural river landscapes can be biodiversity hotspots but are one of the most human altered ecosystems with habitats significantly damaged around the world, and a third of fish populations threatened with extinction. While riparian ecosystems have been negatively altered by anthropogenic activities, effective planning and restoration strategies can reverse negative impacts by improving habitat quality. However, restoring rivers requires appropriate data on current riparian health while also considering priorities for different stakeholders. To address this, a Geographic Information System (GIS) was used to create a new and transferable restoration priority model based on a section of the river Linth in Switzerland as a case study. The restoration priority model is founded on connectivity, river condition, national priority species and species hotspots. Landscape change of the riparian zone was analyzed using aerial imagery and landscape metrics. Almost a quarter of rivers within the study area were considered high or very high restoration priority, with many aquatic species set to benefit from restoration. From 1946 to 2019, the riparian landscape became highly fragmented due to significant growth in impervious surfaces and a concomitant loss of agricultural land. The GIS model provides a tool by which environmental agencies can manage natural features over large scales, while also planning priorities and targeting conservation strategies to the areas of greatest need.

## Introduction

The ecology and biodiversity of river systems offer many benefits but are impacted by anthropogenic activities that decrease hydro-ecological diversity (Janssen et al. 2021). Natural river landscapes are biodiversity hotspots (Tomscha et al. 2017) which are among the most affected by human actions (Revenga et al. 2000). Human activities such as river channelization and regulation, dams, gravel mining, deforestation, and agriculture have changed the biological condition of rivers (Maass et al. 2021). Climate change is also impacting significantly on freshwater species (Griffith and Gobler 2020) as increased water temperature modifies basal metabolic functioning (Pörtner and Farrell 2008), timing of pivotal biological events (Asch 2015), and species occurrence (Harley et al. 2006). Freshwater habitats have become the most damaged in the world (Piczak et al. 2023), with almost a third of fish populations threatened with extinction (WWF 2021).

One of the most significant zones for biodiversity is the riparian zone. Such zones represent a critical interface between terrestrial and aquatic ecosystems which provide significant ecological services (ES) such as eliminating pollutants from run-off, reducing flood damage, increasing fish biodiversity and abundance and delivering animal and plant resources which support recreation and improve human quality of life. Riparian zones are regarded as critical to the preservation of the biological conditions of rivers (Naiman et al. 2000) and landscape change in these areas has a significant impact on the ability of species to function (Stoffers et al. 2022). Therefore, it is essential to have accurate data from which to quantify change in riparian zones and effectively target conservation and protection measures.

### Management of Riparian Zones

There is an urgent need to develop approaches for evaluating ecological riparian zones from multiple viewpoints (Fernandes et al. 2011), allowing integration of complex and unpredictable changes brought on by climate change and human activity. This requires a multidisciplinary approach with extensive datasets for successful conservation (Pohl and Hirsch Hadorn 2008). Spatial data, Geographic Information System (GIS), and landscape metrics are increasingly used for evaluation of strategies for detecting priority sites for restoration. Fernandes et al. (2011) emphasized the use of different landscape metrics to describe the structure of riparian zones along four tributaries of the River Tagus in Portugal. Metrics enabled fragmented areas to be quickly identified, allowing critical areas to be identified for future conservation efforts. In North Central Texas, Atkinson et al. (2010) developed a GIS model whereby riparian zones were ranked in terms of priority and subsequently targeted for restoration work. Etter et al. (2020) highlighted the potential of GIS-based restoration models to efficiently target resources to areas of greatest need, and to minimize impact on other land uses. The model was able to integrate valuable ecological data, and refine previous conservation models and effectively identified priority areas across a large spatial area.

Ecological restoration has emerged as a critical strategy for reducing, and responding to, environmental deterioration caused by global urbanization (Stoffers et al. 2022). River restoration can improve river ecology, prevent additional biodiversity loss and restore lost ecosystem services (Palmer et al. 2005). Lu et al. (2019) conducted a meta-analysis of fifty-five studies to identify the value of river restoration and found that ecological condition was significantly enhanced, particularly in areas affected by “hydromorphological degradation or land-use changes” (p. 30). Furthermore, stream restoration can improve water quality by attenuating dissolved pollutants such as nitrogen (Johnson et al. 2015).

In general, river restoration describes modification for improved hydrological, hydromorphological, or ecological processes of a river section (Wohl et al. 2015). However, restoration does not need to create the former known condition. The decision of what prior state the restoration should be based on can be controversial (Van Diggelen et al. 2001) and the exact former conditions are usually unknown factors (Wohl and Merritts 2007). Wohl et al. (2015) outlined several common river restoration goals (Table 1).

**Table 1:** Summary of the restoration aims of rivers/creeks (adapted from Wohl et al. 2015)

Goal	Description
Aesthetics/recreation/education	Activities that improve community value such as: safety, knowledge. usage, attractiveness and accessibility.
Bank stabilization	Practices aimed at limiting or eliminating bank material erosion or slumping into the river channel - stormwater management is excluded.
Channel reconfiguration	Channel geometry, planform, and/or longitudinal profile changes, as well as daylighting (converting pipes or culverts to open channels).
Dam removal/retrofit	Dams and weirs are removed, as well as repairs and retrofits to existing dams, to mitigate negative repercussions; dam modifications undertaken only to promote fish passage are excluded.
Fish passage	Physical removal of obstructions to upstream/downstream migration of fishes; includes the establishment of alternate channels and migratory barriers installed at crucial points along streams to keep unwanted species from accessing upstream areas.
Floodplain reconnection	Practices that increase the frequency, amplitude, or duration of flooding in floodplain areas, as well as the flow of organisms and materials between channels and floodplain areas.
Flow modification	Practices that modify the frequency and quantity of water delivery (not including stormwater management); often, but not always, connected to impoundment releases and constructed flow controls.
Land acquisition	Practices that secure leases/titles/easements for streamside land with the aim l of preserving or removing affecting agents and/or facilitating future restoration projects.
Instream habitat improvement	Increasing the availability and variety of habitat for target organisms through modifying structural complexity, as well as providing breeding habitat and protection from disturbance and predation.
Instream species management	Practices that directly influence the distribution and abundance of aquatic native species by introducing (stocking) or translocating animal and plant species and/or eliminating invasive species; excludes physical manipulations of habitat/breeding area.

While there may be multiple goals for river restoration, setting priorities can be based on ease of implementation and a lack of resistance rather than biodiversity goals. Studies have identified a range of priorities that are not necessarily aligned with biodiversity gains such as conformity with funders, community acceptance and simplicity of execution (Kondolf et al. 2008). Logar et al. (2019) suggested that setting restoration priorities can offer many benefits that outweigh the costs when taking environmental, social and economic benefits into consideration.

Within Europe, the Water Framework Directive (WFD) sets priorities for all Member States to protect and restore water bodies to ensure healthy aquatic ecosystems for both wildlife and human needs. While the WFD encourages an integrated approach to river management, true integration has been difficult due to time pressures, different government priorities and competing sectors (e.g., energy, aquaculture), which have often led to substandard ecological status (Carvalho et al. 2018). Similar policies, such as the Habitats Directive, focus on river catchments and surrounding land cover types. As such, river network restoration models are needed that can operate at large spatial scales while enabling the integration of data in terms of water quality and land cover/land use. Such models enable variables to be selected and evaluated by a range of stakeholders ranging from government departments to local community groups (Basak et al. 2021). Furthermore, analysis of change over time along riparian zones is critical, particularly in light of rapid urbanization and intensive agriculture (Fernandes et al. 2021a).

### Project Aim

The Linth plain is managed by three different states which border the river system. Each state is responsible for their own river/creek restoration, which implies that the river network is not analyzed as an entire system. The aim of the study was to produce a management tool which could be used by states to identify where greatest restoration priority is possible across the entire river system, regardless of administrative zones. The main objectives of this study were to (i) quantify the landscape pattern along the river system and (ii) propose river and creek sequences for restoration. The developed river network restoration model was based on weighting factors taking the distribution of species hotspots, national priority species, river condition and river connectivity into account and so is highly transferable.

## Methods

### Study Area

**Fig. 1:**
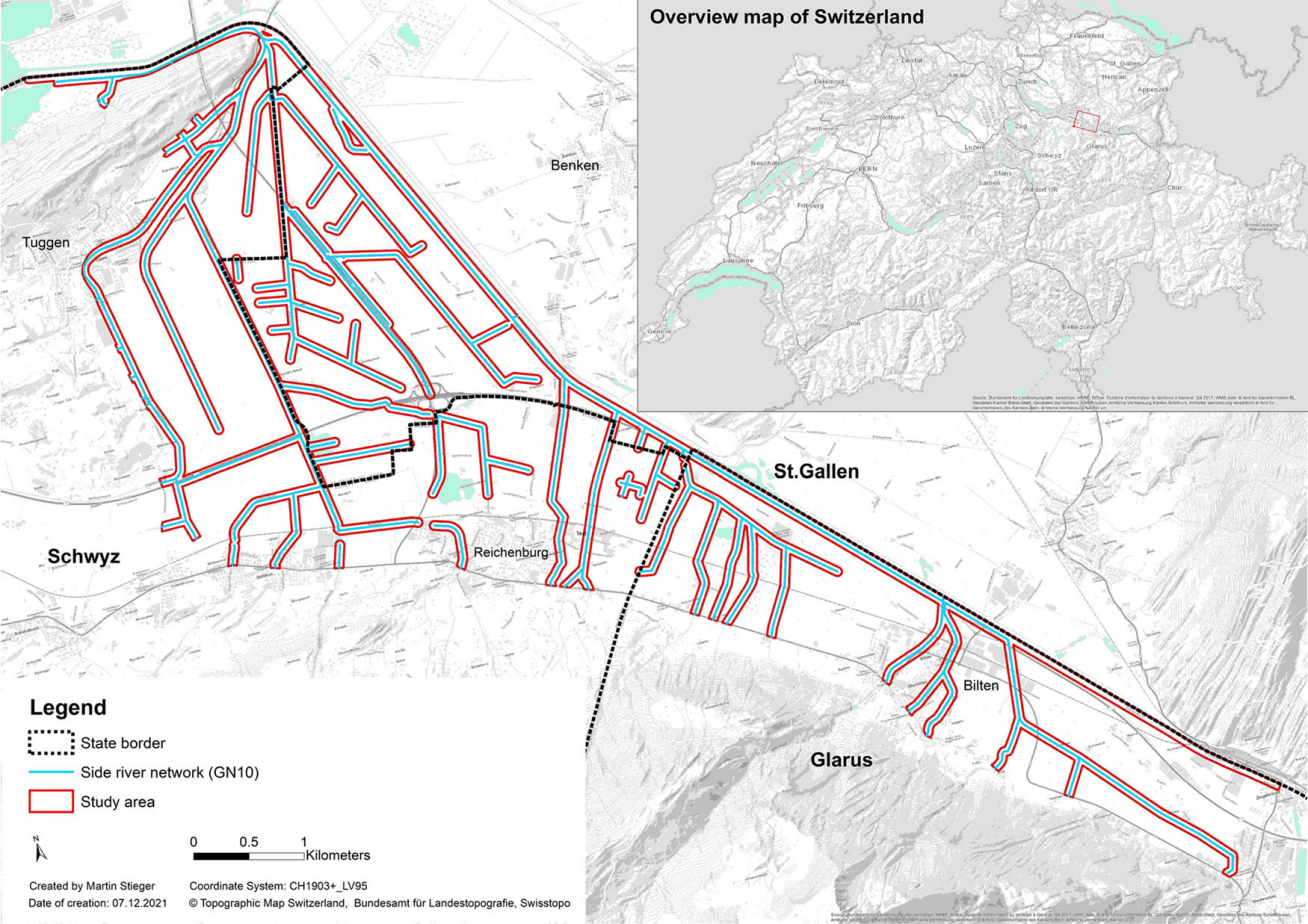
The study area is within a flat area called the Linth plain. The focus lies within the river networks which are within the borders of three different states (St. Gallen, Glarus and Schwyz)

The study was undertaken on the southern river network of the Linth, which is located between the lake of Zurich and Walensee in the east of Switzerland, and the overall catchment has an area of 5.7 km^2^ (Fig. 1). This entire river network south of the river Linth is managed by three different states (St Gallen, Glarus and Schwyz), and has tributaries of different ecological value with restored and non-restored river sections. The area needs additional restoration coordination due to different states sharing their border within the side river network. The area around the tributaries of the Linth are dominated by agricultural land. The study area contains the riparian zone and was created by buffering the main river Linth by 60 m and the side river networks including the ditches with a buffer of 40 m. The buffer distance of 40–60 m and its land cover types was chosen as the most relevant for rainfall-runoff of nutrients and the prevention of sudden pollutant inputs into rivers and creeks (Duan et al. 2021). The study area has experienced significant urbanization over time and the river network has been channelized which is typical for many low lying areas within Switzerland. The research is applicable to river networks which are impacted by increased urbanization, intensive agriculture and hard engineering such as canalization.

The river was strongly altered through a large-scale project to increase agricultural productivity between 1941 and 1964 by draining wetlands, consolidating property, increasing population and road accessibility, as well as channelization of rivers and creeks. For more recent environmental planning, the state authorities in Switzerland are responsible for the administration of water bodies and restoration projects (Kurth and Schirmer 2014). The Swiss Federal Act on the Protection of Waters (SFAPW) was revised in the year 2011 and requested states to restore and reconnect canals and protect natural water habitats. On a national level, the revised SFAPW is essential for the conservation and promotion of biodiversity. In addition, the restoration of water bodies is prioritized, and rivers and creeks should be barrier free, making fish migration possible (Göggel 2012). Furthermore, as noted by Logar et al. (2019), Switzerland has goals to restore a substantial number of river sections by 2090. At present, around 50% of Swiss rivers and creeks below 600 meters (Above Sea Level) are in an unnatural condition (Bammatter et al. 2015) which can be defined by artificial and semi-natural Ecomorphological Condition (EC).

### Land Cover Digitizing Process

Aerial images from 1949, 1983/1984 and 2019 were digitized to provide consistent land cover maps. Image resolution increased with 1949 having a spatial resolution of 1 m, 1984 with 0.5 m and 2019 with 0.1 m. The aerials were all captured between May and June when vegetation growth occurs (Swisstopo 2022). Digitization was performed using ESRI ArcGIS 10.5.1 and vector data assigned the Swiss coordinate system (CH1909_ + LV95).

Five land cover types were defined (Herzog et al. 2001; Table 2) within the study area: Ecological Infrastructure (EI), Forest, Farmland, Settlement and Traffic Infrastructure (TI). The specific digitization rules were developed during the digitization process of all three orthophotos. These rules ensured that land cover types were meaningful to a range of stakeholders while also being generic and comparable to other studies and regions (Gu et al. 2019; Xu et al. 2022).

**Table 2:** LCT and their general digitizing rules

Land cover type	Description
Farmland	Areas which are mainly used for food production such as crops or meadows for animals such as: • Arable land • Tree nursery • Grassland (permanent grassland, periodically flooded grassland, grassland in crop rotation and meadows for farm animals)
Forest	Tree covered areas such as: • Deciduous forest • Coniferous forest • Mixed forest
Settlement	Settlements including green areas surrounding them such as: • Rural settlement and Urban settlement • Farm Building and other single standing buildings (including bunkers) • Park, gardens and backyards • Sites for sports or recreation • Parking lots next to buildings • Outdoor storage place and gravel pits
Traffic infrastructure (TI)	All infrastructures for pedestrians or essential for traffic such as: • Railway, multi-track / single-track • Highway including median strip • Main road • Side street (also farmland patch made from gravel) • Main path/Byway/Farm track and footpath • Parking lots directly next to the road not in a settlement
Ecological infrastructure (EI)	Land cover which are beneficial for biodiversity in a landscape such as: • Fruit tree alley and single standard trees such as fruit trees • Tree row • Ditch/brook (including dry ditch) • Rivers including rocks at the shore and Reed belt at the shore • Lake/pond • Hedgerow and Bushland (including Trees and bushes next to the river shore)

The Minimum Mapping Unit was 50 m^2^ for all land cover types except for forests which needed to fulfill specific requirements given by the states - features smaller than 50 m^2^ were digitized in the land cover in which it was located. The Minimum Mapping Unit was based on the minimum size of a hedgerow to fulfill the size criteria of a hedgerow (Department of Landscape and Nature 2014). Furthermore, the digitization rules were developed by taking size and width suggestions of the states’ rules into account. The rules for digitizing ‘Forest’ (area and width) were based on the state St. Gallen forest definition rule. Creeks smaller than 1 m were also digitized at 1 m due to the spatial resolution of 1946 AI (1 m). This was also done for other features such as roads, tracks, etc. The land cover data were assessed for errors by using basic quality check-ups and applying Topology Rules (Martinez-Lario et al. 2017).

### Landscape Metrics

Landscape metrics were calculated using the Vector-based Landscape Analysis Tools Extension for ArcGIS 10 (Lang and Tiede 2003). Several metrics were chosen for each individual land cover type in 1946, 1984 and 2019 (Table 3). These metrics provided quantitative measures of landscape structure and change over time, offering insights into fragmentation, connectivity and habitat quality within the riparian zone (McGarigal and Marks 1995).

**Table 3:** Definition of the relevant LM (McGarigal and Marks 1995)

Landscape metric index	Range / Unit	General description
Number of patches (NP)	NP > = 1, without limit / None	NP = 1 when the landscape contains only 1 patch of the corresponding patch type
Mean patch size (MPS)	Range: MPS > 0, without limit /Hectares	Equals the sum of the areas (m^2^) of all patches of the corresponding patch type divided by 10,000 (convert to hectares)
Edge density (ED)	ED > = 0, without limit / meter per hectare.	Equals the sum of the lengths (m) of all edge segments involving the corresponding patch type, divided by the total landscape area (m^2^), multiplied by 10,000 (convert to hectares).
Mean nearest neighbor distance (MNN)	MNN > 0, without limit / meter	Equals the sum of the distance (m) to the nearest neighboring patch of the same type, based on nearest edge-to-edge distance, for each patch of the corresponding patch type, divided by the number of patches of the same type
Mean shape index (MSI)	Range: MSI > = 1, without limit / None	Equals the sum of the patch perimeter (m) divided by the square root of patch area (m^2^) for each patch of the corresponding patch type, adjusted by a constant to adjust for a circular standard (vector) or square standard (raster), divided by the number of patches of the same type

### River Network Restoration Model

Channel sequences with high restoration priority would bring the most potential benefits for aquatic river biodiversity, based on national data such as the presence of National Priority Species (NPS) and High Diversity of Aquatic Species (HDAS), river condition, lack of obstacles and connectivity of the river network along with data on sections recently restored. The Federal Office for the Environment (FOEN) designed restoration guidelines which includes HDAS and NPS that have to be considered for restoration plans (Schmidt and Fivaz 2013). Furthermore, producing positive outcomes for biodiversity is an important goal of the strategy (Göggel 2012), thus resembling EU policies such as the Water Framework Directive. The developed river network restoration model is based on the ecomorphological condition, river connectivity (fish migration) and the presence of priority species and high species diversity (Table 4). These data were georeferenced secondary data (vector data) provided by different authorities.

**Table 4:** Data description and weighing factors of the restoration priority (RP) model

Data minimum and maximum points	Weight in priority model	Influence on priority model / weighting factors
River barrier/obstacles: 0 (min) to 2 (max) points	25%	River sequence contains a barrier= • YES = High need for restoration = 2 points • NO = No need for restoration = 0 points
Ecomorphological condition (EC) of the riverbed = natural, semi natural, artificial 0 (min) to 2 (max) points	25%	• “Natural EC” (Muhar et al. 2013: EC = High and EC = Good) = no need for restoration = 0 points • “Semi natural EC” (Muhar et al. 2013: EC = Moderate) = moderate need for restoration = 1 point • “Artificial EC” (Muhar et al. 2013: Poor and EC = Bad) = high need for restoration = 2 points
National priority species (NPS) = NPS River sequences (data relating to fish, amphibia, crabs, mollusks, odonata, ephemeroptera, plecopteran trichobtera and beetles) 0 (min) to 2 (max) points	25%	NPS river sequence= • YES = Lower need to restore NPS sequences due to their existing environment quality = 0 points • NO = Moderate need for restoration due to many absent species = 1 point River sequence borders directly to a NPS (NPS = YES) river sequence: • YES = Higher need for restoration due to benefit for species survival, creating additional habitat and potential spawning grounds (Capne and Kushlan 1991). = 2 points
High diversity of aquatic species (HDAS) (‘hotspot sequences’) 0 (min) to 2 (max) points	25%	HDAS river sequence= • YES = Lower need to restore HDAS sequences due to their existing environment quality = 0 points • NO = Moderate need for restoration due to many absent species = 1 point River sequence borders directly to a HDAS river sequence: • YES = Higher need for restoration due to benefit for species survival, creating additional habitat and potential spawning grounds (Capne and Kushlan 1991). = 2 points

River obstacles (fish migration barriers) show which rivers/creeks are connected and which contain barriers. The reconnection of channels and barrier removal for fish passages is considered a common restoration goal (Table 1) and is a requested condition by the SFAPW. Moreover, Göggel (2012) describes river barrier data as important to evaluate the need for restoring a river sequence. Consequently, the restoration priority is higher if a river sequence contains a barrier (see Table 4).

The ecomorphological condition of a channel sequence provides information about the riverbed and bank slope condition. Moreover, the dataset is considered as base information for strategic restoration planning and delivers significant evidence about how beneficial a restoration project is. Thus, the need of restoration is high if the ecomorphological condition of a river is artificial/non-natural, and low if it is considered natural (Göggel 2012; Table 4).

The FOEN provides secondary data on river sequences which are valuable for biodiversity in terms of the occurrence of species. In addition, there are data on river sequences which are valuable because of the presence of national priority species (NPS) or are showing a high diversity of aquatic species (HDAS) (Schmidt and Fivaz 2013). River sequences with a high number of species/rare species are of high protection status as high biodiversity generally indicates intact ecosystems (Prendergast et al. 1993). Thus, sequences with existing high species diversity and priority species are considered as a lower restoration priority, though their status needs to be protected.

Reduced habitat size is a threat to the existence of aquatic species due to the loss of ecosystem stability and living space (Capne and Kushlan 1991). The likelihood of a local extinction is increased by the disconnection of habitats and accessibility to refugia due to the loss of suitable rescue effects (Stoffers et al. 2022) given by the ability of avoiding unsuitable environmental conditions (Davey and Kelly 2007) or negative impacts (predation and competition) of other species (Zeug et al. 2005). Consequently, if a river sequence is beside another river sequence which has high biodiversity or rare species, there is a higher need to restore the nearby river sequences. Having a river section restored near sequences with priority species or high diversity would give species a greater chance to migrate and gain additional habitats and spawning ground in a bordering channel sequence. The goal is to increase the ecosystem stability and living space. Due to the different migration distances between species, a minimum length of 500 m was chosen to define areas adjacent to priority species or high diversity hotspots (Muhar et al. 2007).

**Fig. 2:**
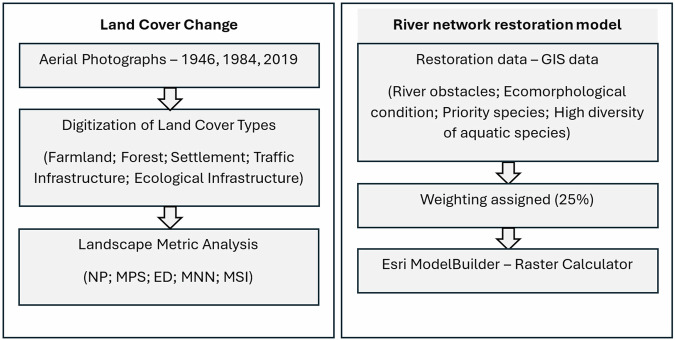
Methodology for quantifying land cover change and development of river network restoration model

The river restoration model was developed using equal weighting of 25% for river obstacles, ecomorphological condition, priority species (NPS) and high diversity of aquatic species (HDAS). However, weights can be applied in consultation with local experts, stakeholders, and policymakers through processes such as the Analytic Hierarchy Process (AHP) or Multi-Criteria Decision Analysis (MCDA). Fernandes et al. (2021b) identify that weights should be assigned by experts based on longitudinal water quality inspections and weights should be tailored to the study site. Ruangpan et al. (2021) also identify that assigning weights should be conducted using a large sample of stakeholders in order to reduce bias while capturing the needs and priorities of different groups. Assigning equal weights of 25% in this study provides a base model which can be updated by stakeholder engagement in future research (Fig. 2).

Weighting factors are assigned as two points for high restoration priority, one point for moderate restoration priority and zero points for no restoration priority. One exception is the river barrier dataset which had its point range simplified by omitting points with a moderate restoration priority. Table 4 was used to calculate the priorities of the river restoration.

The input data were prepared and parameters of the rasterization process based on Ghanem (2017). The reclassification was according to the restoration priority workflow (Table 4). Additionally, to correctly combine queries in the Raster Calculator, all input rasters had the same cell size and position. Work processes were performed using Esri ModelBuilder. The restoration priority model was calculated utilizing the raster calculator in ArcMap with river sequences receiving eight points classed as very high restoration priority, while sequences with lower points (e.g., 0-1 points) classed as very low restoration priority.

## Results

### Land Cover Types

**Fig. 3:**
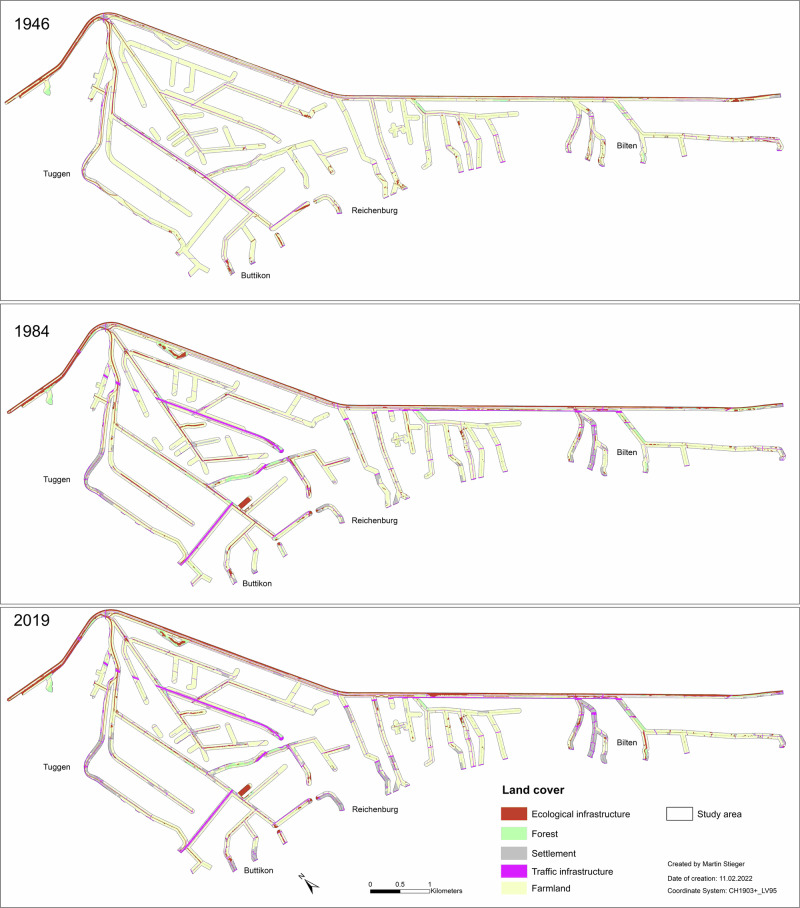
Land cover map of 1946, 1984 and 2019 for the side river network of the Linth. The applied digitization rules are according to different land cover types

The result of the land cover digitization is shown in Fig. 3 with main data presented in Tables 5, 6.

**Table 5:** Land cover categories in each study year

Land cover category	1946		1984		2019	
	Area (km^2^)	*Area (%)*	Area (km^2^)	*Area (%)*	Area (km^2^)	*Area (%)*
Ecological infrastructure (EI)	0.51	8.92	0.67	11.72	0.68	11.95
Forest	0.05	0.93	0.20	3.58	0.16	2.78
Settlement	0.06	0.98	0.28	4.85	0.43	7.52
Traffic infrastructure (TI)	0.24	4.19	0.45	7.84	0.50	8.72
Farmland	4.85	84.98	4.11	72.01	3.94	69.03

**Table 6:** Land cover category change over time

Land cover category	1946–1984		1984–2019		1946–2019	
	Area (km^2^)	*Area (%)*	Area (km^2^)	*Area (%)*	Area (km^2^)	*Area (%)*
Ecological infrastructure	0.16	2.8	0.01	0.2	0.17	3.0
Farmland	−0.74	−13.0	−0.17	−3.0	−0.91	−15.9
Forest	0.15	2.7	−0.05	−0.8	0.11	1.8
Settlement	0.22	3.9	0.15	2.7	0.37	6.5
Traffic infrastructure	0.21	3.6	0.05	0.9	0.26	4.5

Increases in impervious surfaces is the main trend with both Settlement (6.5%) and TI (4.5%) increasing over the last 73 years (Tables 5 and 6). In all years, Farmland dominated the study area followed by EI, although the amount of Farmland decreased (15.9%), largely due to growth of Settlements and TI. While Forest represents the smallest land cover type in all years it experienced a small increase (1.8%) from 1946 to 2019 yet declined (0.8%) from 1984 to 2019 (Table 6). EI covered ~9% of the study area in 1946 and 12% in 2019 which shows a small increase over time. Settlements and TI increased, mainly between 1946 and 1984 (3.9% and 3.6% respectively) with smaller increases between 1984 and 2019. The majority of land cover change occurred between 1946 and 1984 (Table 6).

### Landscape Metrics

Landscape metrics were calculated for each land cover type in each study year (Table 7).

**Table 7:** Landscape metrics for each land cover type at class level over study period

Land cover type	Year	Area (%)	MPS	MSI	NP	MNND	ED
Ecological infrastructure (EI)	1946	8.92	0.21	3.1	245	19.9	3188.6
	1984	11.72	0.26	3.2	262	14.7	2531.8
	2019	11.95	0.27	3.9	256	11.3	2455.3
Farmland	1946	84.98	1.14	2.6	425	2.4	767.5
	1984	72.01	0.86	2.5	478	3.5	887.5
	2019	69.03	0.64	2.5	615	3.2	971.0
Forest	1946	0.93	0.2	1.9	27	168.3	1328.6
	1984	3.58	0.27	1.9	76	105.9	1217.0
	2019	2.78	0.26	2.3	62	92.5	1228.3
Settlement	1946	0.98	0.07	1.3	79	164.0	1525.0
	1984	4.85	0.16	1.4	175	100.6	1048.3
	2019	7.52	0.18	1.5	232	78.5	1038.5
Traffic infrastructure (TI)	1946	4.19	0.13	4.3	181	43.1	5332.9
	1984	7.84	0.25	4.0	181	38.4	3737.3
	2019	8.72	0.3	4.0	168	40.0	3488.7

#### Ecological Infrastructure

Both the area and MPS of EI increased over time, with MPS for EI increasing by 28.6% within the entire study time (0.21 ha to 0.27 ha). When considered alongside the increase in NP from 1946 to 1984 it suggests the addition or creation of EI patches within the study area. The decrease in MNND between EI patches in each study year shows that patches are closer together over time with greater coalescing of patches, also suggested by a decrease in the ED value over time. EI has the second highest MSI value of all five LCT which suggests that EI patches are complex patches which increase in complexity over time.

#### Farmland

This LCT dominated the study area in each year although the class area and MPS decreased, mainly between 1946 and 1984 (0.28 ha) with a smaller change (0.22 ha) between 1984 and 2019. The NP increased over time, specifically between 1984 and 2019, suggesting that Farmland decreased and became more fragmented across multiple, smaller patches. This is also supported by the increase in ED over time with greatest increase between 1946 and 1984 (120 m/ha). Overall, it appears that Farmland experienced the greatest change across all metrics between 1946 and 1984 with some stabilization between 1984 and 2019 although there was some further loss and fragmentation during that period.

#### Forest

While Forest represents the smallest land cover type in all years, both the total area and MPS of Forest increased between 1946 and 2019 with greatest increase occurring between 1946 and 1984. There was a decrease of 20% in Forest between 1984 and 2019 (0.20 to 0.16 km^2^) alongside a small reduction in MPS (0.27 to 0.26 ha). The Number of Patches (NP) increased from 1946 (27) to 1984 (76) followed by a small decrease in 2019 (62). Both MNND and ED decreased from 1946 to 1984 suggesting greater homogenization of forest patches with time. MSI for Forest was lower than Farmland in each year suggesting relatively simple patches with little complexity. The MSI for Forest was second lowest of all five LCT and was only lower than Settlements.

#### Settlement

Settlement increased in total area, MPS and NP in each year studied. This suggested that new patches of Settlement were created over time and existing patches grew. The area, MPS and NP showed the strongest increase between 1946 and 1984. ED and MNND decreased for Settlement suggesting Settlement patches got closer over time and less fragmented with time. The decline in MNND in each study year suggested that patches became closer over time. The MSI values for Settlement were the lowest of all five LCT.

#### Traffic Infrastructure

TI was similar to Settlement with an increase in total class area and MPS over time though NP was stable between 1946 and 1984 and decreased from 1984 to 2019. ED was the highest for TI and experienced the greatest decrease between 1946 and 2019 of all five LCT. MSI was also highest for TI, suggesting this LCT had greatest complexity of all five LCT.

### River Network Restoration Model

The results of the river network restoration model are based on the weighted factors applied in the GIS (Table 4; Fig. 3). The side river network has a total length of 84.5 km within the study area. Only 0.9% (0.8 km) of the analyzed creeks/rivers show a “very high” restoration priority with most of the river system (36.2%, 30.6 km) declared as “moderate”, followed by 23.3% (19.7 km) classed as “high” restoration priority category. Furthermore, 10.8%, (9.1 km) is within the “low” and 5.7% (4.8 km) is within the “very low” restoration priority category. A similar number of rivers belong to the category “unknown” (11.1 km, 13.1%) and the “already restored” rivers (8.5 km, 10.1%). The “unknown” priority category consists of creek and river sequences where secondary data were unavailable.

**Fig. 4:**
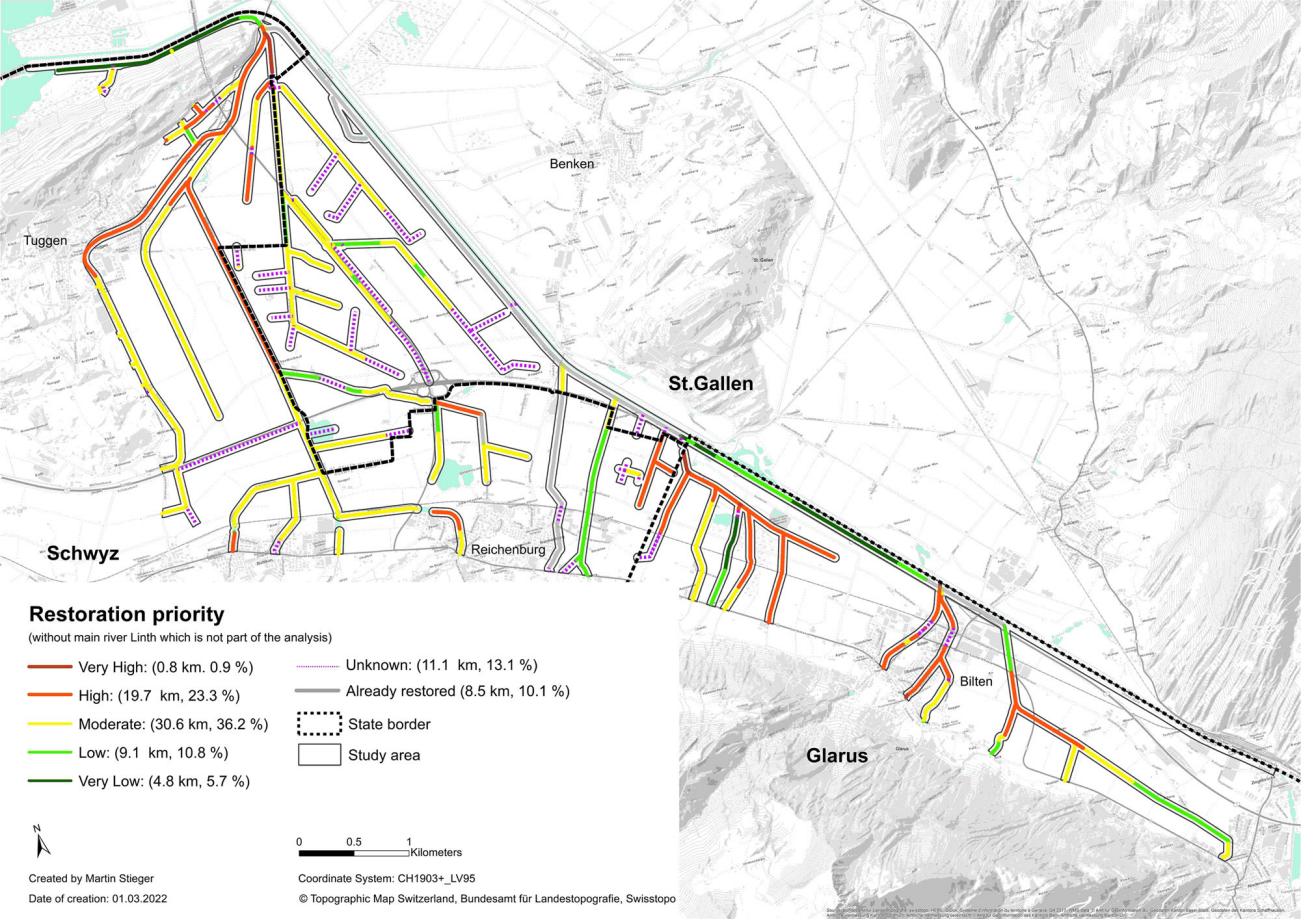
RP for the side river network of the Linth

**Fig. 5:**
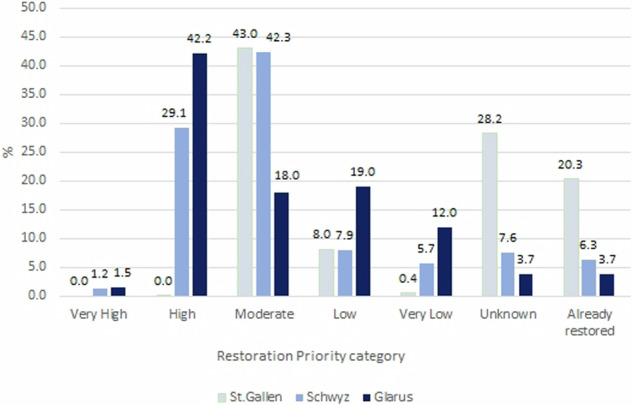
Restoration priority of the river network for each state as a proportion of each state’s river length. (River network length: St. Gallen = 26.8 km; Glarus = 22.08 km; Schwyz = 35.58 km)

The restoration priority results were calculated for each state as a proportion of the state’s river length in the study area (Fig. 4). Glarus showed the smallest number of restored rivers (3.7%) while St. Gallen, with 20.3%, had the highest amount. There are low levels of “very high” restoration priority in Schwyz (1.2%) and Glarus (1.5%). The river network of St. Gallen shows an absence of “very high” and “high” priority sequences. The river network of St. Gallen contains a larger number of rivers of “unknown” sequences (28.2%) while in Glarus only 3.7% of the rivers are within the “unknown” category. A large number of rivers have a “high” restoration priority status in Glarus (42.2%) and Schwyz (29.1%). Moreover, in Glarus and Schwyz a large number of rivers belong to the “moderate” category (43.0% and 43.2% respectively). Glarus has the highest amount of “low” (19%) and “very low” (12%) restoration priority categories (Fig. 5).

#### River Network and Adjacent Land Cover Types

Within the 40 m buffer of priority river sequences, Farmland dominated followed by Ecological Infrastructure. When combined, Settlement and Traffic Infrastructure occupied a larger percentage than Ecological Infrastructure while Forest occupied the lowest proportion of buffered river sequences.

Within ‘very high’ and ‘high’ priority river sequences, Farmland dominated though Forest (8.8%) and Ecological Infrastructure (12%) were present in both categories. Forest mainly occurred in the ‘very high’ priority stretches though there was a similar amount of Forest in the ‘very low’ priority buffer (7.9%). Ecological Infrastructure mainly occurred in ‘low’ (11%) or ‘very low’ priority buffers (32.9%), though almost 20% of Ecological Infrastructure occurred in ‘high’ or ‘very high’ buffers. There tended to be higher amounts of Settlements in ‘high’ or ‘very high’ priority buffers (18.5%) though Traffic Infrastructure had greater presence in ‘low’ or ‘very low’ priority buffers (24.2%).

## Discussion

### Land Cover Change—National and Local Trends

While similar trends occur at both the national level and study area, distinct differences occur within the study area. National data from 2018 confirms Farmland as the dominant land cover (47%) though it declined (17.5%) between 1947 and 2018, similar to trends in the study area (15.9%). Nationally, Forest covers around 24% though occupies only a small proportion (2.4%) of the study area. Between 1984 and 2019, Forest declined in the study area though grew (1.4%) nationally (FSO 2021a). A significant driver of Forest loss within the study area was removal for infrastructure projects and Settlement. Between 1985 and 2018, 10% of newly built land (Settlement and Traffic Infrastructure) occurred on previously forested land (FSO 2021a). Some Forest may have changed to Ecological Infrastructure because of methodological decisions, particularly if Forest area decreased and was below the Minimum Mapping Area (State St. Gallen 2019). Within the riparian study area, Ecological Infrastructure occupied 10.8%, reflecting coverage at the National level (9%). Nationally, Settlement and Traffic Infrastructure occupied 20% within areas of similar altitude (FSO 2021a) with a 1.9% growth rate (1985–2018), which was lower than in the riparian study area (3.6% increase). The construction of a large highway in the study area cannot explain the higher urban growth rate as it was completed in 1973 (Krähenbühl et al. 2006). The proximity of the study area to large urban centers such as Zurich have led to a significant increase in domestic properties.

While area and percentage cover are important, landscape metrics provide greater insights into landscape patterns and changes that may occur over time (Stoffers et al. 2022; Locke 2024). Over time the study area experienced greater fragmentation, mainly because of increased urbanization and an associated loss of farmland. Fagan et al. (2005) emphasized that one of the main reasons for local and regional species disappearance in freshwater ecosystems is habitat fragmentation. Gu et al. (2019) identify that fragmentation has a strong negative effect on water quality over time. Moreover, density and diversity indices such as NP and ED can quantify landscape heterogeneity while also providing insights into water quality (Gu et al. 2019; Xu et al. 2022).

Increased fragmentation in this study, particularly between 1946 and 1984, can partly be explained by the highway construction in 1973 (Krähenbühl et al. 2006) which divided the plain in two parts (Fig. 3). Roads not only act as barriers to animal movement, but cause habitat loss, degradation and road traffic causes significant pollution of nearby streams and waterways (Awonaike et al. 2022). Large scale urbanization in the study area, and nationally, is likely to lead to increased fragmentation (Theodorou 2022; Uuemaa et al. 2007) and associated pressure on natural ecosystems through human activities. Xu et al. (2022) identified a strong positive correlation between water pollution and urban land in the Poyang Lake basin of China. However, Locke (2024) identifies that the relationship between Land Use Land Cover and water quality is complex and must take account of spatial scale and landscape configuration.

While Farmland occupied the greatest proportion of the study area, both its composition and configuration experienced significant change which may impact on river restoration. The metrics identify greater fragmentation of Farmland over time which is widely associated with lower water quality, particularly in riparian zones, due to increased use of fertilizers and pesticides (Xu et al. 2022; Gu et al. 2019).

The small, complex nature of Forest patches in the riparian zone indicate increased threat of removal over time (Arellano-Rivas et al. 2017). The conversion of Forest to urban land between 1984 and 2019 is likely to have led to greater risk of flooding, run-off, soil loss (Atkinson et al. 2010), decreased bank stability (Kauffman et al. 1997) and water quality (Karakus 2020). Studies have found that riparian forests have a considerable role to play in improving water quality (Gu et al. 2019; Karakus 2020; Tolkkinen et al. 2021) and their fragmentation can cause degradation of the biological condition of rivers over time (Yirigui et al. 2019). Consequently, in this study, it is likely that water quality decreased because of increased riparian forest fragmentation, which also increases the priority for restoration. There is a clear need to reduce fragmentation of riparian zones over time to improve the biological condition of the river system (de Mello et al. 2022). Additionally, for slow flowing and small to medium sized rivers like in the Linth plain, the shade produced by riparian vegetation plays an important role by reducing temperatures and balancing out negative effects on the river ecosystems (Ghermandi et al. 2009). Stoffers et al. (2022) also highlight the importance of inter-connected habitats and heterogenous riparian habitats when restoring rivers, particularly for enhanced fish reproduction.

The study area in general is likely to benefit from wide scale uptake of riparian buffer zones, both within urban and agricultural landscapes. While Farmland has decreased over time, it still represents the largest land cover category. Farmers could be incentivized to plant trees and protect rivers and streams through buffer strips (Van Looy et al. 2013). Before planting, the status of riparian plant communities must be monitored, and a strategy developed. Coordination of these schemes is critical and mapping systems and landscape metrics can target initiatives to areas at greatest need (Locke 2024). Prioritizing areas in a coherent fashion can lead to the creation of ‘green veins’ (Grashof-Bokdam and van Langevelde 2005) or wildlife corridors (Polasky et al. 2008) along river networks, thus improving water quality but also enhancing local biotic communities (Logar et al. 2019) and providing an opportunity for species migration, particularly considering climate change. Bryant (2006) declared riparian zones as ecologically valuable corridors that connect isolated patches and counteract fragmentation.

### River Network Restoration Model

River restoration has become a priority, and a legal requirement for many European nations (Carvalho et al. 2018), including Switzerland, which is relevant for water management by government departments (Swiss Federal Council 2022). By 2090 Switzerland intends to restore 4000 kilometers of their river network (Logar et al. 2019). The river network restoration model identifies river stretches that would benefit aquatic species through restoration. Considering the high number of endangered freshwater species (Janssen et al. 2021) and the increasing stressors from climate change (Griffith and Gobler 2020), the river network restoration model provides state authorities with important data from which targeted measures can be implemented.

Within the study area almost a quarter of rivers and creeks have a “high” (23.3%) or “very high” (0.9%) restoration priority. The model is based on the aquatic species which were in the national database of priority species and high diversity data. Within the study area, river sequences with priority species and high diversity included species of dragonflies, fish, aquatic moss and crabs. When restoring river sequences, specific target species and their habitat preferences must be taken into consideration (Zingraff-Hamed et al. 2018).

This research highlights inter-state variations in restoration priority mapping with Glarus and Schwyz showing significant need to restore river sequences while St Gallen has lower potential (Fig. 5). However, St. Gallen already has a high number of rivers that were already restored within the study area. This study also identifies that spatial data both between states and across countries needs to be harmonized and of high quality. St. Gallen had a high number of rivers within the “unknown” category. Secondary data about ecological connectivity had several river sequences unavailable in all three states. Consequently, the river and creeks without connectivity data have “unknown” priorities due to the lack of valuable input data about current river conditions. Analysis of the missing river sequences highlighted that unknown sections were either underground or open creeks. Muhar et al. (2013) identified ecologically sensitive river stretches in the Alpine Arc, spanning Switzerland, Austria, Germany, France and Slovenia and identified that Swiss data on ecological connectivity were often incomplete (40% of Swiss rivers) and that the coverage level varied between different states. This highlights the potential for GIS and restoration models to harmonize data collection methods and share best practice across European countries. These improvements are also relevant for the federal and state’s authorities, especially for St. Gallen, which shows the highest level of connectivity incompleteness within the study area.

The developed restoration priority model takes failures of past restoration projects into account. It is known that river restoration projects were unsuccessful due to having no re-colonization potential of aquatic species, which can be explained by the shortage of source populations and a high amount of migration barriers (Stoll et al. 2014; Stoffers et al. 2022). While the source population was indicated by priority and high diversity data for each river sequence, migration barriers were included to consider the possibility of re-colonization within the river network. The outcome of the river network restoration model relies on the timeliness and general quality of the input data. Therefore, states and responsible government agencies are advised to keep the data (e.g., ecomorphological river condition, river barriers, priority species and high diversity sequences) as current as possible.

It is unrealistic to assume that all river segments can be prioritized for restoration due to financial constraints and competing land use requirements. However, by developing a coordinated model across multiple states, there is greater potential for environmental gains and improved water quality (Marchese 2015). The developed restoration model would encourage expansion of river sequences which have high diversity of aquatic species and national priority species, thus reducing unfavorable environmental conditions (Davey and Kelly 2007) and other stressors such as competition and predation by other species (Zeug et al. 2005). Therefore, increasing bushland and forest in riparian zones and restoring the river network of “very high” and “high” priority sequences would be beneficial for many aquatic species.

#### River network and adjacent land cover

While riparian restoration is an increasing priority in the context of climate change (Seavy et al. 2009), restoration is also complicated by climate change (Harris et al. 2006; Palmer et al. 2008). In order to successfully restore rivers and creeks it is essential to consider the stressors for aquatic species. Griffith and Gobler (2020) found that stressors for freshwater species increased due to climate change while the impact on aquatic species are complex due to the interaction of multiple stressors. Furthermore, ecosystem warming increases stress for freshwater species (Griffith and Gobler 2020) due to the modified timing of pivotal biological events (Asch 2015), changed basal metabolic functioning (Pörtner and Farrell 2008) and altering species occurrence (Harley et al. 2006). These findings have important implications for restoration models as adjacent land cover types can be integrated and evaluated according to the ecological services they provide, such as reduced nutrient run-off, vegetation shading and improved water quality (Van Looy et al. 2013). The river network restoration model did not take account of adjacent land cover types and focused solely on aquatic species, river connectivity and ecomorphological condition. Integration of land cover types within riparian buffer zones would enable models to be developed further with priorities being assigned to more vulnerable sequences. Within the study area, all river sequences had low cover of Ecological Infrastructure and Forest within the riparian buffer (Table 8) which indicates considerable potential to increase vegetation in future restoration projects (Tolkkinnen et al. 2021). Settlements dominate along river sequences with “high” restoration priority, pointing to the need to include local residents within restoration projects. Åberg and Tapsell (2013) studied the long-term social benefits of the rehabilitation of the river Skerne in Darlington, United Kingdom. The study suggested that the river restoration enhanced the quality of life in the local community, providing well-used recreational space with appealing green space and wildlife that people could enjoy. Smith et al (2014) calls for a ‘blended’ approach, with both ecological and social objectives being considered in river restoration projects. Indeed, Basak et al. (2021) identify that while economic and ecological issues still govern river restoration projects, social benefits must increase in prominence in future river restoration research.

**Table 8:** Area (%) of each land cover type within the riparian zone (40 meter buffer)

Land cover type (2019)	Very high priority	High priority	Moderate priority	Low priority	Very low priority
Ecological infrastructure (EI)	12.0	7.6	7.5	11.0	32.9
Farmland	65.6	70.7	72.7	65.1	45.9
Forest	8.8	2.1	2.8	5.6	7.9
Settlement	5.4	13.1	6.9	7.2	0.2
Traffic infrastructure (TI)	8.2	6.5	10.1	11.1	13.1

### Recommendations for Future River Network Restoration Models

Atkinson et al. (2007) developed management and protection strategies of the watershed of the Lewisville lake in Texas and suggested that the restoration and protection of healthy riparian zones is an alternative to river restoration if financial resources are limited when planning restoration projects. Consequently, future models should account for the ecological services of adjacent land cover. For instance, river sequences that are dominated by Farmland or Settlements would have greater need for restoration than river sequences which consist of Ecological Infrastructure or Forest.

Future research should consider changes in water quality, aquatic condition and diversity alongside mapping changes to the river network and adjacent land cover over time. This study focused on mapping river change and land cover change over time. However, temporal data relating to water quality and biodiversity are important to identify how riparian changes impact on water quality and aquatic species.

The use of high resolution data, both temporally and spatially, is invaluable in creating river restoration models (Locke 2024). Indeed, directives such as the Water Framework Directive and the Habitats Directive, have led States to collect a wide range of data that can be implemented in restoration models across Europe. When coupled with earth observation data, accurate spatial models can be developed to assess the relationship between Land Use Land Cover and water quality (Locke 2024). These models can serve as a base from which changes can be monitored over time using metrics on landscape configuration and water quality (Apan et al. 2002). Fernandes et al. (2011) used a range of landscape metrics to quantify the pattern of riparian vegetation along the river Tagus in Portugal and suggested that combining composition metrics (e.g., mean patch size) with configuration metrics (e.g., nearest neighbor) can effectively characterize the landscape structure and improve river management. Additionally, Paterson et al. (2019) emphasized that landscape metrics can be used to propose and design new habitats and are not restricted to analyzing existing land cover.

This study presents two complementary approaches based on mapping land cover change over time and the creation of a river network restoration model. Mapping land cover change is possible globally due to wide availability of high-resolution imagery from both aircraft and satellites. The methodology also presents a transferable and consistent method for digitizing land cover types at a high-spatial resolution, thus enabling changes in composition and configuration to be quantified using landscape metrics. While this study focused on riparian zones, expansion to include land cover change at a catchment scale is advocated to ensure that the “cumulative effects” of land cover change is fully understood (Locke 2024, p. 5). Furthermore, data on water quality should be integrated alongside longitudinal studies on land cover change. Large scale directives, such as the Water Framework Directive, have led to many States collecting water quality data over time. The integration of these datasets can yield new insights into the relationship between land cover change, land use and water quality.

The river network restoration model is based on a relatively small group of variables which are widely available. The simplicity of the model enables adoption by planners and policy makers, while more complex models demand diverse datasets which in turn require careful management and processing which can be time-consuming (Locke 2024). While equal weights are employed in this study, the approach enables multiple stakeholders to inform weights and make alterations if necessary. GIS plays a critical role in the entire process, not only in terms of mapping land cover change, but also in terms of integrating spatial data, enabling weights to be applied based on stakeholder engagement, identifying river sequences that should be prioritized for restoration and even the design of new landscapes that can enhance water quality and riparian habitats (Paterson et al. 2019; Thomas et al. 2020).

## Conclusion

The riparian zone within the study area experienced fragmentation over the last 73 years though there was greater change between 1946 and 1984 than between 1984 and 2019. Moreover, increased urbanization within the riparian zone of the study area reflected trends at the national level. The amount of forest within the riparian zone decreased slightly between 1984 and 2019, which can lead to a decrease of ecological services such as the prevention of nutrient run-off and a decreased cooling effect through shading. Increased urbanization and concomitant loss of both Forest and Ecological Infrastructure is symptomatic of many river systems. Such riparian change demands stronger protection, restoration and conservation with coordinated decision-making that integrates excellent data with the views of all stakeholders.

Freshwater habitats have become the most damaged in the world, with almost a third of fish populations threatened with extinction. Therefore, the need to quickly counteract and prioritize the most beneficial restoration sequences is significant for species survival. Developing riparian restoration priority models using a range of high resolution spatial data across large spatial scales enables stakeholders to interact and create management plans to protect and enhance vulnerable river sequences. GIS offer significant potential to not only integrate pertinent spatial data, but enable the visualization of priorities while permitting weights to be altered based on stakeholder input and local conditions. The restoration priority model identifies river sequences that offer the greatest benefits for aquatic species through restoration efforts, while mitigating potential stressors such as fragmented habitats, limited accessibility to refugia, unsuitable environmental conditions, and negative impacts from predation and competition by other species.

The developed restoration model can be applied worldwide based on widely available data. Earth observation data offers significant potential for planners to assess water quality, map Land Use Land Cover and monitor change over time. These data in turn can be integrated in a GIS for weighting and stakeholder engagement in order to be tailored to national and local policies. These spatial restoration models promote communication and interaction between agencies, thus promoting care across catchments and mutual benefits for entire river systems.

## Data Availability

Aerial images were sourced from: Swisstopo (2022) Kachelung SWISSIMAGE Zeitreise (Bundesamt für Landestopografie swisstopo). Bern: Swisstopo, Available at: https://map.geo.admin.ch/#/map?lang=de&center=2711138.85,1226274.67&z=5&bgLayer=void&topic=luftbilder&layers=ch.swisstopo.swissimage-product_1946;ch.swisstopo.swissimage-product@year=1983;ch.swisstopo.lubis-luftbilder_schwarzweiss@year=1983;ch.swisstopo.swissimage-product.metadata@year=1983;ch.swisstopo.lubis-bildstreifen@year=all,f;KML%7Chttps://public.geo.admin.ch/gkuVFmsoTkCsOTSH6fAniA&catalogNodes=luftbilder,1180,1186,1179&timeSlider=1983 [Accessed 02 January 2022]. Secondary data on river sequence biodiversity, occurrence of species and presence of national priority species were sourced from the FOEN: Schmidt and Fivaz (2013) Fliessgewässerabschnitte mit hoher Artenvielfalt oder national prioritäre Arten, Grundlagendaten für die Planung von Revitalisierungen (Schlussbericht). Federal Office for the Environment (FOEN), Bern, https://plattform-renaturierung.ch/mediathek/fliessgewaesser-abschnitte-mit-hoher-artenvielfalt-oder-national-prioritaeren-arten/.
